# Supplementary material for: Predictors of Sexual Dysfunction in Veterans with Post-Traumatic Stress Disorder
Source: J Clin Med. 2019 Mar 29;8(4):432. doi: 10.3390/jcm8040432 (PMC6518171; doi:10.3390/jcm8040432)
Supplement: Supplementary file 1 [file jcm-08-00432-s001.zip › Material S1.docx]

Supplementary Materials: Material S1

1.1. Prevalence of Comorbid Disorders and Drug Use and Association with SD

1.1.1. Psychiatric Comorbidity and Psychotropic Drugs

According to results obtained by M.I.N.I., the most common comorbid psychiatric disorders among veterans with PTSD in our sample are current and lifetime major depressive episode (MDE) (overall 54.6%) and panic disorder (current and lifetime 26.3%). Current suicidality (low suicide risk) is present in 18 (6%) veterans. At least one of other anxiety disorders is present in 61 (20.3%) participants (agoraphobia 10, social phobia 8, obsessive-compulsive disorder 4 and generalized anxiety disorder 44). Thirteen (4.3%) have AUD and 7 (2.3%) have a drug related disorder in the last year. Three participants are classified with antisocial personality disorder and one with an eating disorder. None of the participants are classified with a current psychotic disorder although 3 have lifetime psychotic disorder.

Based on number of events and relevancy for our study, following psychiatric disorders were tested for differences in provisional sexual dysfunction diagnosis: MDE current, MDE lifetime, suicidality (low risk), panic disorder current and lifetime, other anxiety disorders and AUD. Veterans with provisional diagnosis of SD are significantly more likely to have current MDE, lifetime panic disorder and an AUD (Table S1.)

Twenty (6.7%) participants do not use any psychotropic drugs. Average number of psychotropic drugs is 2.49 (median = 2). Fourteen percent take one drug, 29.4% two drugs, 28.8% three drugs and 21.1% four or five psychotropic drugs in combination. The most common psychotropics are anxiolytics with 237 (79%) participants taking at least one anxiolytic therapy. 199 (66%) participants use at least one antidepressant, 94 (31%) of them use at least one hypnotic and sedative, 92 (31%) use at least one antiepileptic and 74 (25%) at least one antipsychotic. Only 3 participants use drugs used in addictive disorders (methadone). Differences in use of groups of psychotropic drugs are presented in Table 2. Only antidepressants differ significantly depending to SD status. Veterans with provisional diagnosis of sexual dysfunction are significantly more likely to use antidepressant compared to veterans without provisional SD diagnosis.

1.1.2. Other diseases and drugs

The most common self-reported non-psychiatric diseases in the sample are diseases of the circulatory system (n=137, 45.7%) followed by endocrine, nutritional and metabolic diseases (n=64, 21.3%) and diseases of the musculoskeletal system and connective tissue (n = 64, 20.6%). When it comes to diseases already recognized as significantly related to sexual dysfunction, the most common self-reported in our sample are essential hypertension which affects 128 (42.7%) veterans with PTSD, followed by DM (n=42, 14%), diseases of lipoprotein metabolism (n=23, 7.7%) and hyperplasia of prostate 10 (3%). Some of the conditions known to influence SD are rarely present in our sample: sleep apnea 4, malignant neoplasm of testis 2, epilepsy 2, other hypothyroidism 2, multiple sclerosis 2, Parkinson disease 1, stroke 1 and autoimmune thyroiditis 1.

**Table S1.** Comorbidity and drugs and differences according to presence of sexual dysfunction.

| ***At Least One of*** | **All**  ***N* = 300** | **SD**  **No** | **SD**  **Yes** | **Statistics** | **Probability** |
| --- | --- | --- | --- | --- | --- |
| MDE current | 76 (25.3%) | 18 (25%) | 54 (75%) | 4.366 | 0.037 |
| MDE lifetime | 103 (34.3%) | 30 (30.9%) | 67 (69.1%) | 1.150 | 0.284 |
| Suicidality (low risk) | 15 (5%) | 4 (30.8%) | 9 (69.2%) | 0.114 | 0.736 |
| Panic disorder, current | 31 (10.3%) | 6 (20.7%) | 23 (79.3%) | 2.960 | 0.085 |
| Panic disorder, lifetime | 48 (16%) | 10 (20.8%) | 38 (79.2%) | 5.197 | 0.023 |
| Other anxiety disorders | 61 (20.3%) | 16 (28.6%) | 40 (71.4%) | 1.321 | 0.250 |
| Alcohol use disorders | 13 (4.3%) | 0 | 10 (100%) | 5.616 | 0.018 |
| Antidepressants | 146 (48.7%) | 41 (29.3%) | 99 (70.7%) | 4.205 | 0.040 |
| Antipsychotics | 66 (22%) | 23 (34.8%) | 43 (65.2%) | 0.003 | 0.957 |
| Antiepileptics | 92 (30.7%) | 26 (30.2%) | 60 (69.8%) | 1.306 | 0.253 |
| Anxiolytics | 237 (79%) | 73 (33.5%) | 145 (66.5%) | 1.176 | 0.278 |
| Hypnotics and sedatives | 94 (31.3%) | 26 (31%) | 58 (69%) | 0.918 | 0.338 |
| Diabetes mellitus | 42 (14%) | 15 (39.5%) | 23 (60.5%) | 0.365 | 0.546 |
| Disorders of lipoprotein metabolism | 23 (7.7%) | 3 (14.3%) | 18 (85.7%) | 4.328 | 0.037 |
| Hypertension, essential | 130 (43.3%) | 39 (32%) | 83 (68%) | 0.949 | 0.330 |
| Hyperplasia of prostate | 10 (3.3%) | 2 (20%) | 8 (80%) | 1.041 | 0.308 |
| Antihypertensives | 91 (30.3%) | 28 (32.6%) | 58 (67.4%) | 0.360 | 0.549 |
| Drugs used in diabetes | 16 (5.3%) | 8 (53.3%) | 7 (46.7%) | 2.306 | 0.129 |
| Lipid modifying agents | 31 (10.3%) | 6 (20%) | 24 (80%) | 3.375 | 0.066 |

Differences in occurrence of essential hypertension, DM, disorders of lipoprotein metabolism and hyperplasia of prostate were tested against the presence of SD diagnosis. Veterans with disorders of lipoprotein metabolism are significantly more likely to have SD diagnosis compared to their counterparts. Even tough 8 out of 10 participants with hyperplasia of prostate have SD, the difference is not significant. There is no significant difference in frequency of DM and essential hypertension compared to SD diagnosis (TableS1).

The most common self-reported medication is related to cardiovascular therapy, followed by medications for musculoskeletal system. When it comes to drugs previously recognized as related with SD, the most common is antihypertensive therapy with 91 (30.3%) participants using at least one antihypertensive. At least one lipid modifying agents therapy is used by 31 (10.3%) participants, drugs used in diabetes by 16 (5.3%), H2 blockers and antihistamines by 5, corticosteroids for systemic use and immunosuppressants and drugs for erectile dysfunction by only one participant.

Due to number of events, only use of antihypertensives, drugs used in diabetes and lipid modifying agents are compared to SD diagnosis. Veterans with provisional diagnosis of SD are significantly more likely to use lipid modifying agents compared to veterans without SD diagnosis (Table S1.)
