# Supplementary material for: Predictors of Sexual Dysfunction in Veterans with Post-Traumatic Stress Disorder
Source: J Clin Med. 2019 Mar 29;8(4):432. doi: 10.3390/jcm8040432 (PMC6518171; doi:10.3390/jcm8040432)
Supplement: Supplementary file 1 [file jcm-08-00432-s001.zip › Table S2.docx]

**Table S2.** Correlation coefficients.

|  | **Erectile Function** | **Orgasmic Function** | **Sexual Desire** | **Intercourse Satisfaction** | **Overall Satisfaction** | **Pre-Mature Ejaculation** |
| --- | --- | --- | --- | --- | --- | --- |
| Age | −0.008 | −0.018 | −0.010 | −0.056 | −0.003 | 0.117 |
| Elementary education | −0.012 | −0.027 | 0.016 | −0.044 | −0.058 | −0.012 |
| Secondary education | 0.029 | 0.104 | 0.053 | 0.094 | 0.077 | 0.031 |
| Higher education | −0.028 | −0.119* | −0.098 | −0.085 | −0.034 | −0.086 |
| Low income | −0.190** | −0.172** | −0.164** | −0.137** | −0.157** | 0.039 |
| Medium income | 0.177** | 0.161** | 0.176** | 0.156** | 0.158** | −0.040 |
| High income | 0.026 | 0.020 | −0.044 | −0.058 | −0.012 | 0.007 |
| Not married | −0.158** | −0.111 | −0.087 | −0.115* | −0.039 | −0.179** |
| Divorced | −0.099 | −0.104 | 0.013 | −0.116* | −0.116 | −0.078 |
| Married^1^ | 0.184** | 0.148** | 0.039 | 0.174** | 0.087 | 0.154* |
| In relationship | 0.350** | 0.250** | 0.190** | 0.344** | 0.222** | 0.160** |
| MDE, current | −0.131* | −0.125* | −0.080 | −0.146* | −0.133* | .098 |
| MDE, lifetime | −0.045 | −0.024 | −0.009 | −0.043 | −0.019 | −0.009 |
| Suicidality (low risk) | −0.02 | −0.00 | −0.03 | 0.07 | 0.08 | 0.12 |
| Panic disorder, current | −0.018 | −0.004 | −0.032 | −0.072 | −0.083 | 0.119 |
| Panic disorder, lifetime | −0.130* | −0.126* | −0.115 | −0.090 | −0.126* | 0.037 |
| Other anxiety disorders | −0.086 | −0.096 | −0.079 | −0.116* | −0.156** | 0.037 |
| Alcohol use disorders | −0.113 | −0.046 | −0.154** | −0.112 | −0.129* | 0.077 |
| Diabetes mellitus | 0.044 | 0.080 | 0.091 | 0.055 | 0.117 | 0.148* |
| Hypertension (essential) | −0.147* | −0.161* | −0.066 | −0.092 | −0.124* | 0.062 |
| Hyperplasia of prostate | −0.128* | −0.131* | −0.079 | −0.135* | −0.180** | 0.077 |
| Dis. of lipoprotein metabolism^2^ | −0.089 | −0.118* | −0.096 | −0.111 | 0.018 | 0.098 |
| Antidepressants | −0.139* | −0.178** | −0.152** | −0.095 | −0.064 | 0.006 |
| Antipsychotics | −0.07 | −0.06 | 0.01 | 0.01 | .03 | −0.09 |
| Hypnotics and sedatives | −0.117* | −0.119* | −0.064 | −0.097 | −0.114 | −0.064 |
| Anxiolytics | −0.06 | −0.01 | −0.01 | −0.05 | −0.05 | −0.02 |
| Antiepileptics | −0.01 | −0.01 | −0.04 | −0.01 | −0.00 | 0.08 |
| Antihypertensives | −0.097 | −0.057 | −0.024 | −0.061 | −0.086 | 0.016 |
| Drugs used in diabetes | −0.023 | −0.049 | −0.040 | −0.064 | 0.051 | −0.046 |
| Lipid modifying agents | −0.033 | −0.031 | 0.000 | −0.016 | −0.021 | 0.042 |
| Cluster B symptoms | −0.102 | −0.059 | −0.051 | −0.056 | −0.088 | 0.048 |
| Cluster C symptoms | −0.077 | 0.022 | −0.114 | .073 | −0.048 | 0.100 |
| Cluster D symptoms | −0.305** | −0.259** | −0.307** | −0.291** | −0.300** | 0.185** |
| Cluster E symptoms | −0.214** | −0.171** | −0.173** | −0.206** | −0.233** | 0.125* |
| Total PTSP symptoms | −0.261** | −0.200** | −0.239** | −0.237** | −0.257** | −0.159** |
| War deployment *(in months)* | 0.138* | 0.106 | 0.034 | 0.178** | 0.131 | 0.093 |
| Relationship satisfaction | 0.354** | 0.349** | 0.253** | 0.431** | 0.507** | 0.045 |

* *p* < 0.05; ** *p* < 0.01; ^1^ married and in cohabitation; ^2^ Disorders of lipoprotein metabolism.
