# Supplementary material for: Predictors of Sexual Dysfunction in Veterans with Post-Traumatic Stress Disorder
Source: J Clin Med. 2019 Mar 29;8(4):432. doi: 10.3390/jcm8040432 (PMC6518171; doi:10.3390/jcm8040432)
Supplement: Supplementary file 1 [file jcm-08-00432-s001.zip › Table S3.docx]

**Table S3.** Summary of the final step in hierarchical regression analysis for the overall sample.

|  | **Erectile Function** | | | **Orgasmic Function** | | | **Sexual Desire** | | | **Intercourse Satisfaction** | | | **Overall Satisfaction** | | | **Premature Ejaculation** | | |
| --- | --- | --- | --- | --- | --- | --- | --- | --- | --- | --- | --- | --- | --- | --- | --- | --- | --- | --- |
|  | B | SE | β | B | SE | β | B | SE | β | B | SE | β | B | SE | β | B | SE | β |
| Age | −0.07 | 0.10 | −0.04 | −0.01 | 0.03 | −0.01 | −0.01 | 0.02 | −0.02 | −0.07 | 0.05 | −0.08 | 0.01 | 0.02 | 0.02 | 0.08 | 0.05 | 0.09 |
| Higher education |  |  |  | −0.94 | 0.67 | −0.08 |  |  |  |  |  |  |  |  |  |  |  |  |
| Low income | −−2.79 | 3.11 | −0.13 | −1.27 | 1.07 | −0.18 | 0.13 | 0.81 | 0.02 | 0.70 | 1.34 | 0.07 | −0.23 | 0.79 | −0.04 |  |  |  |
| Medium income | −0.90 | 3.03 | −0.05 | −0.74 | 1.05 | −0.11 | 0.13 | 0.79 | 0.10 | 1.21 | 1.30 | 0.12 | 0.10 | 0.79 | 0.02 |  |  |  |
| Not married | 0.56 | 2.16 | 0.02 |  |  |  |  |  |  | 1.30 | 1.49 | 0.09 |  |  |  | −1.87 | 1.19 | −0.11 |
| Divorced |  |  |  |  |  |  |  |  |  | 0.58 | 1.47 | 0.04 |  |  |  |  |  |  |
| Married ^1^ | .88 | 1.67 | .03 | −0.01 | 0.49 | −0.1 |  |  |  | 0.79 | 1.22 | 0.07 |  |  |  | 0.05 | 0.89 | 0.01 |
| In relationship | 7.69 | 2 | **0.28**** | 1.98 | 0.66 | **0.21*** | 0.93 | 0.40 | **0.13*** | 3.94 | 0.97 | **0.30**** | 1.17 | 0.44 | **0.15*** | 1.99 | 1.10 | 0.13 |
| MDE, current ^2^ | 0.76 | 1.36 | 0.03 | 0.23 | 0.45 | 0.03 |  |  |  | 0.02 | 0.66 | 0.01 | 0.22 | 0.35 | 0.04 |  |  |  |
| Panic dis. Lifetime ^3^ | −2.03 | 1.47 | −0.08 | −0.99 | 0.49 | −0.11 |  |  |  |  |  |  | −0.71 | 0.38 | −0.11 |  |  |  |
| Other anxiety dis. ^4^ |  |  |  |  |  |  |  |  |  | −0.6 | 0.69 | −0.05 | −0.60 | 0.36 | −0.10 |  |  |  |
| Alcohol use dis. ^5^ |  |  |  |  |  |  | −1.62 | 0.69 | **−0.13*** |  |  |  | −1.47 | 0.71 | **−0.12*** |  |  |  |
| Diabetes mellitus |  |  |  |  |  |  |  |  |  |  |  |  |  |  |  | 1.71 | 0.85 | **0.12*** |
| Hypertension, esse. ^6^ | −3.99 | 1.14 | **−0.20**** | −0.98 | 0.38 | **−0.15*** |  |  |  | −1.49 | 0.55 | **−0.15**** | −0.71 | 0.28 | **−0.14*** |  |  |  |
| Hyperplasia prost. ^7^ | −1.16 | 2.95 | −0.02 | −1.13 | 1.02 | −0.06 |  |  |  | −1.04 | 1.44 | −0.04 | −1.53 | 0.76 | −0.11 |  |  |  |
| Dis. of lipoprotein metabolism ^8^ |  |  |  | −1.09 | 0.71 | −0.08 |  |  |  |  |  |  |  |  |  |  |  |  |
| Antidepressant | −1.3 | 1.22 | −0.06 | −1.12 | 0.38 | **−0.15**** | −0.71 | 0.28 | **−0.14*** |  |  |  | −0.36 | 0.28 | −0.07 |  |  |  |
| Hypnotics and sed. ^9^ | −0.71 | 1.18 | −0.03 | −0.13 | 0.41 | −0.02 |  |  |  |  |  |  |  |  |  |  |  |  |
| War deployment ^10^ | 0.02 | 0.03 | 0.04 |  |  |  |  |  |  | 0.02 | 0.01 | 0.08 |  |  |  |  |  |  |
| Cluster B symptoms | 0.18 | 0.21 | 0.06 | 0.06 | 0.07 | 0.06 | 0.05 | 0.05 | 0.07 | 0.15 | 0.10 | 0.10 | 0.06 | 0.05 | 0.08 | −0.09 | 0.11 | −0.06 |
| Cluster C symptoms | 0.30 | 0.41 | 0.05 | 0.27 | 0.14 | 0.12 | 0.02 | 0.10 | 0.01 | 0.10 | 0.20 | 0.03 | 0.10 | 0.10 | 0.06 | 0.10 | 0.23 | 0.03 |
| Cluster D symptoms | −0.46 | 0.15 | **−0.24**** | −0.15 | 0.05 | **−0.23**** | −0.14 | 0.04 | **−0.29**** | −0.23 | 0.07 | **−0.25**** | −0.11 | 0.04 | **−0.23**** | 0.21 | 0.08 | **0.21*** |
| Cluster E symptoms | −0.27 | 0.19 | −0.11 | −0.05 | 0.07 | −0.06 | −0.01 | 0.05 | −0.01 | −0.15 | 0.01 | −0.08 | −0.07 | 0.05 | −0.11 | 0.05 | 0.10 | 0.04 |
|  | R^2^=0.257  Adjusted *R*^2^ = 0.208  Df 1,2 = 16, 247  *F* = 5.651, *p* < 0.001 | | | *R*^2^ = 0.248  Adjusted *R*^2^ = 0.194  Df 1,2 = 17, 239  *F* = 4.628, *p* < 0.001 | | | *R*^2^ = 0.166  Adjusted *R*^2^ = 0.135  Df 1,2 = 10, 276  *F* = 5.482, *p* < 0.001 | | | *R*^2^ = 0.251  Adjusted *R*^2^ = 0.204  Df 1,2 = 16, 272  *F* = 5.714, *p* < 0.001 | | | *R*^2^ = 0.210  Adjusted *R*^2^ = 0.165  Df 1,2 = 15,278  *F* = 4.854, *p* < 0.001 | | | *R*^2^ = 0.116  Adjusted *R*^2^ = 0.085  Df 1,2 = 9,269  *F* = 3.778, *p* < 0.001 | | |

* *p* < 0.05; ** *p* < 0.01; ^1^ married and in cohabitation; ^2^ Major depressive episode, current; ^3^ Panic disorder lifetime; ^4^ Other anxiety disorders; ^5^ Alcohol use disorders; ^6^ Hypertension, essential; ^7^ Hyperplasia of prostate; ^8^ Disorders of lipoprotein metabolism; ^9^ Hypnotics and sedatives; ^10^ in months; significant values are in bold.
