# Supplementary material for: Predictors of Sexual Dysfunction in Veterans with Post-Traumatic Stress Disorder
Source: J Clin Med. 2019 Mar 29;8(4):432. doi: 10.3390/jcm8040432 (PMC6518171; doi:10.3390/jcm8040432)
Supplement: Supplementary file 1 [file jcm-08-00432-s001.zip › Table S4.docx]

**Table S4.** Summary of the final step in hierarchical regression analysis for the subset sample of veterans in a relationship.

|  | **Erectile Function** | | | **Orgasmic Function** | | | **Sexual Desire** | | | **Intercourse Satisfaction** | | | **Overall Satisfaction** | | | **Premature Ejaculation** | | |
| --- | --- | --- | --- | --- | --- | --- | --- | --- | --- | --- | --- | --- | --- | --- | --- | --- | --- | --- |
|  | B | SE | β | B | SE | β | B | SE | β | B | SE | β | B | SE | β | B | SE | β |
| Age | −0.06 | 0.10 | −0.03 | −0.01 | 0.03 | −0.01 | −0.01 | 0.03 | −0.02 | −0.07 | 0.05 | −0.09 | 0.02 | 0.02 | 0.03 | 0.08 | 0.06 | 0.09 |
| Higher education |  |  |  | −0.59 | .68 | −0.05 |  |  |  |  |  |  |  |  |  |  |  |  |
| Low income | −8.13 | 3.18 | −0.01 | −0..57 | 1.10 | −0.08 | 0.47 | 0.85 | 0.09 | 1.02 | 1.35 | 0.10 | 0.15 | 0.73 | 0.03 |  |  |  |
| Medium income | 0.48 | 3.07 | 0.02 | −0.44 | 1.06 | −0.06 | 0.72 | 0.83 | 0.14 | 1.27 | 1.29 | .13 | .29 | .71 | .06 |  |  |  |
| Not married | 1.91 | 3 | 0.04 |  |  |  |  |  |  | 2.71 | 1.65 | 0.13 |  |  |  | 0.33 | 1.65 | 0.01 |
| Divorced |  |  |  |  |  |  |  |  |  | 1.48 | 1.56 | 0.08 |  |  |  |  |  |  |
| Married ^1^ | −0.22 | 1.76 | −0.01 | −0.51 | 0.49 | −0.06 |  |  |  | 0.76 | 1.18 | 0.06 |  |  |  | 0.42 | 0.10 | 0.03 |
| MDE, current ^2^ | −0.81 | 1.43 | −0.04 | −0.15 | 0.47 | −0.02 |  |  |  | −0.49 | 0.66 | −0.04 | −0.03 | 0.32 | −0.01 |  |  |  |
| Panic dis. Lifetime ^3^ | −0.85 | 1.53 | −0.04 | −0.39 | 0.52 | −0.05 |  |  |  |  |  |  | −0.51 | 0.35 | −0.08 |  |  |  |
| Other anxiety dis. ^4^ |  |  |  |  |  |  |  |  |  | −0.67 | 0.71 | −0.06 | −0.53 | 0.33 | −0.09 |  |  |  |
| Alcohol use dis. ^5^ |  |  |  |  |  |  | −1.52 | 0.77 | −0.11 |  |  |  | −1.22 | 0.70 | 0.09 |  |  |  |
| Diabetes mellitus |  |  |  |  |  |  |  |  |  |  |  |  |  |  |  | 1.91 | 0.89 | **0.14*** |
| Hypertension, esse. ^6^ | −3.08 | 1.17 | **−0.17**** | −0.78 | 0.39 | **−0.12*** |  |  |  | −0.84 | 0.54 | −0.09 | −0.48 | 0.26 | −0.10 |  |  |  |
| Hyperplasia prost. ^7^ | −2.88 | 3.52 | −0.05 | −169 | 1.22 | −0.08 |  |  |  | −2.19 | 1.67 | −0.08 | −1.99 | 0.82 | **−0.13*** |  |  |  |
| Dis. of lipoprotein metabolism ^8^ |  |  |  | −1.04 | 0.71 | −0.09 |  |  |  |  |  |  |  |  |  |  |  |  |
| Antidepressant | −1.17 | 1.26 | −0.06 | −0.96 | 0.39 | **−0.15*** | −0.73 | 0.30 | **−0.15*** |  |  |  | −0.47 | 0.26 | **−0.11*** |  |  |  |
| Hypnotics and sed. ^9^ | −0.6 | 0.03 | 0.04 | −0.05 | 0.42 | −0.01 |  |  |  |  |  |  |  |  |  |  |  |  |
| War deployment ^10^ | 0.03 | 0.03 | 0.06 |  |  |  |  |  |  | 0.02 | 0.10 | 0.07 |  |  |  |  |  |  |
| Cluster B symptoms | 0.18 | 0.21 | 0.06 | 0.04 | 0.07 | 0.04 | 0.04 | 0.05 | 0.05 | 0.10 | 0.19 | 0.07 | 0.03 | 0.05 | 0.04 | −0.12 | 0.12 | −0.08 |
| Cluster C symptoms | 0.02 | 0.40 | 0.03 | 0.22 | 0.14 | 0.10 | −0.01 | 0.10 | 0.01 | −0.20 | 0.07 | 0.02 | 0.01 | 0.09 | 0.01 | 0.15 | 0.24 | 0.05 |
| Cluster D symptoms | −0.38 | 0.14 | **−0.21**** | −0.15 | 0.05 | **−0.24**** | −0.14 | 0.04 | **−0.30**** | −0.11 | 0.09 | **−0.23**** | −0.09 | 0.03 | **−0.19**** | 0.23 | 0.09 | **.23**** |
| Cluster E symptoms | −0.25 | 0.19 | −0.11 | −0.04 | 0.06 | −0.05 | −0.01 | 0.05 | −0.02 | 0.02 | 0.01 | −0.10 | −0.05 | 0.04 | −0.10 | 0.11 | 0.11 | 0.09 |
| Relationship satisf. ^11^ | 0.43 | 0.09 | **0.29**** | 0.14 | 0.03 | **0.28**** | 0.06 | 0.02 | **0.16**** | 0.27 | 0.04 | **0.39**** | 0.16 | 0.02 | **0.44**** | 0.05 | 0.05 | 0.07 |
|  | *R*^2^ = 0.279  Adjusted *R*^2^ = 0.243  Df 1,2 = 17, 207  *F* = 5.457, *p* < 0.001 | | | *R*^2^ = 0,295  Adjusted *R*^2^ = 0,239  Df 1,2 = 16, 203  *F* = 5.310, *p* < 0.001 | | | *R*^2^ = 0.196  Adjusted *R*^2^ = 0.161  Df 1,2 = 10, 276  *F* = 5.227, *p* < 0.001 | | | *R*^2^ = 0.316  Adjusted *R*^2^ = 0.273  Df 1,2 = 16, 250  *F* = 7.382, *p* < 0.001 | | | *R*^2^ = 0.384  Adjusted *R*^2^ = 0.344  Df 1,2 = 15, 232  *F* = 9.651, *p* < 0.001 | | | *R*^2^ = 0.107  Adjusted *R*^2^ = 0.072  Df 1,2 = 9, 235  *F* = 3.019, *p* < 0.001 | | |

* *p* < 0,05; ** *p* < 0,01; ^1^ married and in cohabitation; ^2^ Major depressive episode, current; ^3^ Panic disorder lifetime; ^4^ Other anxiety disorders; ^5^ Alcohol use disorders; ^6^ Hypertension, essential; ^7^ Hyperplasia of prostate; ^8^ Disorders of lipoprotein metabolism; ^9^ Hypnotics and sedatives; ^10^ in months; ^11^ Relationship satisfaction; significant values are in bold.
